# Newborn Screening for Lysosomal Storage Disorders: Methodologies for Measurement of Enzymatic Activities in Dried Blood Spots

**DOI:** 10.3390/ijns5010001

**Published:** 2018-12-21

**Authors:** Michael H. Gelb, Zoltan Lukacs, Enzo Ranieri, Peter C. J. I. Schielen

**Affiliations:** 1 Departments of Chemistry and Biochemistry, University of Washington, Seattle, WA 98195, USA; 2 Newborn Screening and Metabolic Diagnostics Unit, Hamburg University Medical Center, 20246 Hamburg, Germany; 3 Head Biochemical Genetics, Directorate of Genetics & Molecular Pathology, SA Pathology at the Women’s & Children’s Hospital, North Adelaide SA 5006, Australia; 4 Head Reference Laboratory for Neonatal Screening, Center for Health Protection, National Institute for Public Health and the Environment (RIVM), P. O. Box 1, 3720 BA Bilthoven, The Netherlands

**Keywords:** newborn screening, lysosomal storage diseases, cutoff values, tandem mass spectrometry, diagnosis, dried blood spots, enzymatic activity assays

## Abstract

All worldwide newborn screening (NBS) for lysosomal storage diseases (LSDs) is performed as a first-tier test by measurement of lysosomal enzymatic activities in dried blood spots (DBS). The currently two available methodologies used for measurement of enzymatic activities are tandem mass spectrometry (MS/MS) and digital microfluidics fluorimetry (DMF-F). In this chapter we summarize the workflows for the two platforms. Neither platform is fully automated, but the relative ease of workflow will be dependent upon the specific operation of each newborn screening laboratory on a case-by-case basis. We provide the screen positive rate (the number of below cutoff newborns per 100,000 newborns) from all NBS laboratories worldwide carrying out MS/MS-based NBS of one or more LSDs. The analytical precision of the MS/MS method is higher than that for DMF-F as shown by analysis of a common set of quality control DBS by the Centers for Disease Control and Prevention (CDC). Both the MS/MS and DMF-F platforms enable multiplexing of the LSD enzymes. An advantage of MS/MS over DMF-F is the ability to include assays of enzymatic activities and biomarkers for which no fluorimetric methods exist. Advantages of DMF-F over MS/MS are: (1) simple to use technology with same-day turn-around time for the lysosomal enzymes with the fastest rates compared to MS/MS requiring overnight analytical runs.; (2) the DMF-F instrumentation, because of its simplicity, requires less maintenance than the MS/MS platform.

## 1. Introduction

The practice worldwide for newborn screening (NBS) of lysosomal storage diseases (LSDs) is performed by measurement of lysosomal enzymatic activities in dried blood spots (DBS) on newborn screening cards. In the accompanying review article [1], we list the current NBS laboratories performing prospective screening for LSDs and the specific method used. Since the publication of the accompanying chapter, the California NBS laboratory has introduced NBS for LSDs using tandem mass spectrometry (MS/MS). We have summarized the substrates used for each lysosomal enzymatic assay, as well as the NBS performance parameters in terms of general concepts (positive predictive values versus rates of screen positives). We also discuss the issue of normalization of assay cutoff values for the two screening platforms. Finally, we covered the post-analysis of first-tier NBS data, including statistical tools and the use of second-tier biochemical metabolite assays to reduce the number of false positives.

In the current chapter we focus on methodologies used for first-tier NBS of LSDs based on measurement of lysosomal enzymatic activities in DBS. Both MS/MS and fluorimetry (now in digital microfluidics format, DMF-F) are used in NBS programs. 

## 2. MS/MS and DMF-F Workflows

In this section we summarize the workflows for both MS/MS and DMF-F platforms for the processing of DBS for LSD NBS. Neither method is fully automated, but involves a number of manual steps, as well as steps that can be carried out with automated instrumentation. Both platforms start by adding a ~3 mm punch of each DBS to a separate well of a 96-well, microtiter plate. In Table 1, we summarize the steps and number of pipet liquid transfers per 96-well plate for the assay of two LSDs, for example, Pompe and MPS-I, since these are on the Recommended Uniform Screening Panel (RUSP) in the USA. In the case of DMF-F, two microfluidic cartridges are needed since each hold 48 samples. In Table 1, we also give the approximate hands-on time for each step for the MS/MS assay only as the DMF-F assay has not been tested in the author’s laboratories. 

All steps in the DMF-F assay are done on the plate reader except for the initial DBS extraction, which requires a plate shaker. The MS/MS method requires a plate incubator, a plate centrifuge, and a multi-jet type solvent evaporator. Both platforms require a number of manual liquid transfers using multi-channel pipettors, with DMF-F requiring 21 liquid transfers, and MS/MS requiring seven (Table 1). It is not clear which method requires less hands-on time for sample preparation, and the exact full time per sample will depend on the number of samples and the setup of each laboratory. Larger NBS laboratories may choose to use automated liquid pipetting stations, whereas smaller laboratories will likely use hand-operated, multi-channel pipettors. It is fair to say that both methods are very simple to execute.

A key difference is that with DMF-F a separate, pH-optimized buffer is used for each lysosomal enzymatic reaction. The DBS punch is extracted into a buffer, and the extract is split on the DMF-F microfluidics cartridge and mixed with separate assay buffer reagents, one for each enzymatic reaction [2]. In the case of the MS/MS assay, a single 3 mm DBS punch is incubated in a single assay cocktail containing substrates and internal standards for assay of enzymes for NBS of Pompe, MPS-I, Krabbe, Niemann-Pick-A/B, Fabry, and Gaucher LSDs [3]. Essentially all lysosomal enzymes are maximally active within the pH range ~2.8–4.5, and thus the pH is not always at the optimal value for each enzyme in the MS/MS 6-plex assay. Despite the pH not being optimized, high analytical precision is obtained in the MS/MS assays as will be shown below. MS/MS, assay of the additional LSD enzymes (for example, those relevant to LSDs MPS-II, -IIIB, -IVA, -VI, VII, and CLN2) requires a second 3 mm DBS punch [4] because a single acceptable buffer for all 12 enzymes is difficult to achieve and to date has not been reported. A key difference is that multiple incubations per newborn can be combined prior to a single injection into the mass spectrometer instrument [5], whereas with DMF-F, additional microfluidics cartridges and readers will be required if more than four diseases are analyzed per newborn since each cartridge delivers up to four cocktails. 

The original MS/MS assay of LSDs was performed using the method of flow-injection, whereby the sample is directly infused into the ionization source of the mass spectrometry without liquid chromatography [6]. At that time samples were processed by solid-phase extraction through pads of silica to remove buffer salts, which interfere with the ionization process. To avoid the solid-phase extraction step, LaMarca’s and Kasper’s groups introduce a liquid chromatography (LC) column online with the mass spectrometer [7,8]. This LC-MS/MS method was adopted by the Illinois NBS laboratory, and has been used for prospective NBS of six LSDs (Pompe, MPS-I, Fabry, Gaucher, Niemann-Pick-A/B, Krabbe) with MPS-II added in Dec. 2017 (Rong Shao, personal communication). After these developments, Gelb et al. replaced the solid-phase extraction step with a liquid-liquid extraction step using the organic solvent ethyl acetate to capture the enzymatic products and internal standards with the removal of buffer components, and this was followed by MS/MS with flow injection [3]. This is the basis for the NeoLSD kit introduced more recently by PerkinElmer. Flow injection-MS/MS is used by all NBS labs now prospectively screening of LSDs except as noted in the next paragraph. NBS laboratories that screen for amino acids and acyl carnitines use flow injection-MS/MS to analyze for these analytes in DBS. These laboratories have the option to use the same MS/MS equipment for flow injection-MS/MS analysis of a subset of LSDs.

When the MS/MS method is applied to some LSDs (in particular the sulfatase-related disorders, MPS-IVA and MPS-VI), flow injection-MS/MS needs to be replaced with LC-MS/MS. This is because the some of the sulfated substrates can undergo breakdown in the heated electrospray ionization source (in-source breakdown) to the same products that are formed enzymatically. This increases the blank assay response. With LC-MS/MS, the enzymatic substrate and product are separated by chromatography, and only the product signal eluting with the product retention time is integrated. LC-MS/MS is being used in the Washington state NBS laboratory for the pilot study of MPS-II, MPS-IIIB, MPS-IVA, MPS-VI, and MPS-VII (Elliott, S.; Gelb, M.H.; Scott, C.R.; manuscript in preparation), and is used for prospective NBS in the Illinois NBS laboratory for seven LSDs, including MPS-II [9], and in Taiwan for MPS-II and MPS-VI [10]. In the Illinois NBS laboratory, one DBS punch is used for the 6-plex mentioned above, a second punch is used for MPS-II, and the reaction mixtures are combined together for a single 7plex LC-MS/MS run. Flow-injection-MS/MS is sufficient for most LSDs, including all six in the NeoLSD assay kit, since the substrates do not undergo significant in-source breakdown. It is also possible to use a single punch with a single assay cocktail to assay the same seven LSD enzymes screened in Illinois [5].

For the DMF-F method, the measurements of the enzymatic activities for Pompe and MPS-I assays are typically done with an incubation time of ~3 h (which occurs on the microfluidic cartridge in the plate reader) [11]. The original MS/MS method used an overnight incubation because the multiplex panel includes Krabbe disease that is associated with an enzyme that is relatively slow compared to the Pompe and MPS-I enzymes [6,12]. There is currently no DMF-F assay for Krabbe disease. The Missouri NBS laboratory uses DMF-F for Pompe, MPS-I, Gaucher, and Fabry diseases and an overnight incubation for Krabbe disease followed by a fluorimetric reading using a standard 96-well plate reader. Some NBS laboratories choosing not to screen for Krabbe disease have opted to perform MS/MS-based screening for Pompe and MPS-I with a ~3 h incubation, and this is followed by an overnight run on the mass spectrometer. However, DMF-F assays are read relatively quickly after the incubation, whereas the MS/MS analysis typically is done overnight to collect all of the data at about 1–2 min per sample. Thus, with a 3 h incubation, MS/MS data is available in the morning of day 2, whereas DMF-F data is available at the end of day 1 (without the inclusion of Krabbe disease). Most NBS laboratories carry out a series of second-tier testing on first-tier NBS positive DBS prior to the preparation of the NBS report. With this in mind, each laboratory will have to evaluate the requirement for same day enzymatic activity data for Pompe and MPS-I.

Additional LSD enzymes other than the Krabbe enzyme are relatively slow enzymes and will probably require an overnight incubation for MS/MS and fluorimetric assays (MPS-IIIA, MPS-IVA, MPS-VI) [4,13]. In the Taiwan NBS laboratory, all LSD enzyme assays are carried out overnight because of the inclusion of these slow enzymes [10], whereas the Illinois NBS laboratory uses an overnight incubation because of the inclusion of Krabbe disease [9].

## 3. Screen Positive Rates

As previously discussed [1], the positive predictive value is not a valid parameter that can be used at this time to compare results with MS/MS and DMF-F, and the best parameter we have available is the screen positive rate. The latter is defined as the number of below cutoff samples measured in the first-tier NBS analysis per 100,000 newborns. As pointed out previously [1], the greater proportion of attenuated enzymatic activity screen-positive samples are false positives, and thus the screen positive rate is almost equivalent to the false positive rate. Such numbers are equally well available from prospective pilot studies, pilot studies with de-identified DBS, and prospective NBS programs. Accurate determination of the limiting (and thus meaningful) positive predictive value requires that the number of newborns tested is greater than the reciprocal of the disease frequency [1], (often >> 100,000 newborns).

In any consideration of the screen positive rate, one has to report the cutoff value used by each laboratory as the number of positives will go up as the enzymatic activity cutoff value is raised. It is common practice for NBS laboratories to start their program with a conservative cutoff value and adjust the level over time based on a number of factors. NBS laboratories select their cutoffs primarily by measuring the enzymatic activities in a set of DBS from normal neonates and from a set of DBS from patients confirmed to have the LSD. 

**Figure 1:**
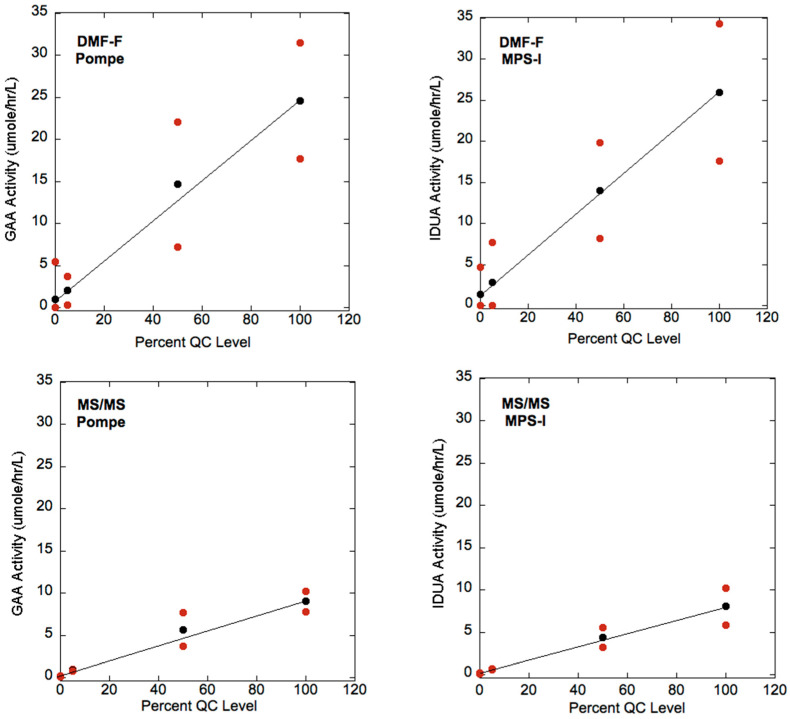
Plots of the precision data taken from the certificate reports for the Centers for Disease Control and Prevention (CDC) quality control DBS for GAA (Pompe disease) and alpha-iduronidase (IDUA, MPS-I) using either DMF-F or MS/MS (reports provided as Supplemental Material). The CDC carried out repetitive assays using either DMF-F or MS/MS with 10 punches from each quality control DBS. Plotted is the mean enzymatic activity (black dots) for the BASE sample (0 % QC level), the LOW sample (5% QC level), the MEDIUM sample (50% QC level), and the HIGH sample (100% QC level). The red dots are the 95% upper and lower confidence intervals.

As a first approximation, a comparison of data using different assays can be performed by taking absolute cutoff enzymatic activities and normalizing to the mean activities among newborns (say the daily mean or the weekly mean or medium). This scaling adjusts for the fact that the two assays use, for example, different substrates and buffer conditions, thus giving different absolute enzymatic activities in units of say mole per hour per liter of blood even if the same sample is analyzed (as is shown in Figure 1). However, as noted in detail in the accompanying article [1], this scaling method is problematic for Pompe disease because of the contribution from off a target enzyme, and the off-target contribution is higher for DMF-F compared to MS/MS [1], Thus, it is clear that one cannot always compare screen positive rates for different assays using the same absolute enzymatic activity cutoff values (scaling problem), and for some assays of LSDs, such as Pompe disease, the use of the same percentage of the population mean or medium cutoff value is also problematic [1]. In cases where there is no off-target enzyme problem, use of percent of mean or medium activities to compare multiple assays is likely to be acceptable.

In Table 2 and Table 3 we give the screen positive rates for all NBS laboratories worldwide who have reported data for MS/MS assays. We also provide the laboratory-chosen cutoff values in terms of percentage of the population mean enzymatic activity. The data comes from the primary literature and also from unpublished data obtained from the director of each NBS laboratory (which was previously presented as a poster at the 2017 APHL NBS conference in New Orleans, Louisiana). All data is from prospective pilot studies or prospective NBS programs except for the data from Washington state, which comes from a pilot study with de-identified samples. 

The New York NBS laboratory does not use a fixed cutoff value, but a dynamic one of 15% of the daily mean GAA enzymatic activity, and has not changed their cutoff since the commencement of their Pompe NBS program (communication from J. Orsini, Wadsworth Laboratory). The Washington NBS laboratory (MS/MS) used the same cutoff as New York (15%). The Ohio NBS laboratory (MS/MS) uses a lower cutoff of 8.5% of mean GAA activity, and in Table 2 we also give the rate had they used 15% of mean activity. The Illinois NBS laboratory (MS/MS) uses a more conservative cutoff of 18% of mean GAA activity, and we also give the rate of screen positives had they used 15% The Kentucky NBS laboratory (MS/MS) does use cutoffs, but rather the CLIR post-analysis computational tools using a panel of six lysosomal enzymes to reduce the number of false positives [14]. The New York and Wisconsin NBS laboratories have started to adopt the CLIR tools to reduce the number of screen positives (conference presentations from J. Orsini in New York and M. Baker in Wisconsin). A detailed outline of the CLIR method is given in the accompanying review article [1].

Table 3 gives worldwide screen positive rates from those NBS programs using MS/MS for MPS-I. In all assays of MPS-I (MS/MS and DMF-F) assay buffers contain adequate amounts of D-saccharic acid-1,4-lactone to fully inhibit the action of beta-glucuronidase on trace amounts of beta-isomer in the synthetic IDUA substrate [1].The use of this inhibitor is required since virtually all preparations of IDUA substrate contain finite contamination from the beta-anomer since the IDUA substrate is made by isomerization of the beta-anomer to the alpha-anomer required by the IDUA enzyme. It seems reasonable that the use of the same percentage of the population mean IDUA activity constitutes an equivalent cutoff value by which to compare all NBS programs for MPS-I. The Illinois NBS laboratory (MS/MS) chooses to use a more conservative cutoff value, but we provide the screen positive rate had they used the cutoff of the other laboratories. Again, the Kentucky NBS laboratory uses CLIR tools rather than a fixed cutoff. The recent data from the prospective pilot study of MPS-I in NY was carried out with a cutoff of 15% of mean IDUA activity. More recently, the NY NBS laboratory has started prospective NBS for MPS-I and has reduced their cutoff to 8%, and the screen positive rate is expected to drop. The Missouri NBS laboratory dropped their cutoff for MPS-I from about 18% of mean IDUA activity to their current value of 7.2% about three years ago.

## 4. Assay Imprecision

There is much discussion of statistical parameters that affect the rate of screen positives. In an upcoming paper we establish that the most important assay metric for affecting the false positive rate is the assay imprecision near the screen cutoff [18]. Imprecision near the cutoff will cause some of the newborns who have an enzymatic activity truly above the cutoff to be measured to lie below the cutoff, and vice-versa. 

The only available precision data measured on an identical set of DBS using MS/MS and DMF-F are the assays performed in the same laboratory at the Centers for Disease Control and Prevention (CDC, Altanta, GA, USA). The CDC performs repeat measurements using 20 DBS punches from each of their Quality Control DBS standards (BASE, LOW, MEDIUM, and HIGH). The HIGH standard is made from 100% pooled blood, whereas the BASE standard is made from 100% leukocyte-depleted, heat-treated blood. The LOW standard is 5% pooled blood, and the MEDIUM standard is 50% pooled blood [19] Certification data using MS/MS and DMF-F were presented at the American Public Health Laboratories NBS meeting in New Orleans 2017, and are available to the public on the CDC web portal (https://www.cdc.gov/labstandards/nsqap_resources.html). Certification reports taken from the web portal for Pompe and MPS-I using DMF-F and MS/MS are given as Supplemental Figures S1 and S2, and Figure 1 gives a graphical representation of the data. Each report gives the mean enzymatic activity and 95% upper and lower confidence intervals for the 20 repeat measurements. From the latter, one obtains the standard deviation in the measurement using the standard textbook formula: Standard deviation = (95% upper confidence limit-mean)/1.96 = (mean-95% lower confidence limit)/1.96.

It is clear from the data in Figure 1 that the assay imprecision is much higher for DMF-F than for MS/MS for all Quality Control DBS standards and at the screen cutoffs.

Note that the absolute enzymatic activities (μmole/h/L) are higher for DMF-F than for MS/MS (Figure 1). This is due to the use of individualized optimized pH buffers for each enzymatic reaction using the DMF-F platform, whereas in the MS/MS method multiple enzymatic reactions are carried out in a single buffer with a compromise in pH (see Section 2). It is not the absolute enzymatic activities that matter, but the imprecision at the screen cutoff that is the important contributor to the screen positive rate [18], and the latter is higher for DMF-F compared to MS/MS (Figure 1).

## 5. Substrate Concentration and the Product Versus Time Progress Curve

For enzymatic reactions, substrate binds to the active site of the enzyme prior to the chemical transformation step. If substrate concentration is high enough (greater than the Michaelis constant characteristic for each enzyme/substrate pair, *K_M_*), all enzyme in the assay will contain bound substrate, and the reaction will not proceed faster if the concentration of substrate is further increased (saturation). In this case the product versus time curve is linear. At lower concentrations of substrate, the enzymatic rate of substrate-to-product conversion will drop as the substrate is depleted, and this will give rise to a fall off from linearity in the product versus time curve if the fraction of substrate consumed is significant compared to the total substrate in the assay. For all LSD enzymatic activity assays reported to date using DBS, the fraction of total substrate consumed at the end of the incubation period is very small (less than 5%). This explains why all reported progress versus time curves for MS/MS-based LSD-enzyme assays are linear despite the fact that in some LSD assays the concentration of substrate in the assay is not sufficient to fully saturate the enzyme [4,6,13,20,21,22,23,24]. The only exception seen so far is the enzyme TPP1, which deviates from linearity after an assay incubation time of about 12 h, due to enzyme instability rather than substrate depletion [6]. The fact that the progress curves are linear also shows that product inhibition under the assay conditions is essentially zero.

## 6. Expansion of Assay Platforms to Additional LSDs

In Table 4 we list all available LSD assays that have been published using either the MS/MS or DMF-F platforms. The enzymatic activity for MPS-IIIA is very low in DBS, and, so far, only an MS/MS assay has been reported for DBS samples, although a standard 96-well fluorimetric assay works for fibroblasts where much larger amounts of enzyme are used [25]. There is a standard 96-well fluorimetric plate assay for Niemann-Pick-A/B, but two assays per newborn are required because of the substantial false negative problem with the fluorimetric substrate [26,27]. The MS/MS assay uses a substrate with a structure closer to the natural substrate and does not lead to this false negative problem [27]. Although there are fluorimetric and MS/MS assays for arylsulfatase A for analysis of metachromatic leukodystrophy, the enzyme is very unstable in DBS, and there is an enormous pseudodeficiency problem [28,29]. NBS of this LSD by LC-MS/MS measurement of accumulated sulfatides in DBS is an option for metachromatic leukodystrophy [30]. A second option may be to measure the abundance of the arylsulfatase A protein by immunoassay (personal communication with D. Matern, Mayo Clinic), but it is possible that false negatives will occur if the mutation affects the enzymatic activity without compromising protein stability. Also, the pseudodeficiency problem is still expected to be a problem with this type of assay. Niemann-Pick-C is due to a deficient lipid transporter, and the only reported NBS assay is a lipid biomarker (bile acid B) measured by LC-MS/MS [31]. The original lysosomal acid lipase assay developed by Hamilton and colleagues uses a non-specific fluorimetric substrate and in the presence and absence of specific inactivator of the lipase, thus requiring two assays per patient [32]. Recently, a new lysosomal acid lipase-specific substrate was developed for a single assay per newborn using either MS/MS or fluorescence [23].

The use of LC coupled to MS/MS does not constitute a large increase in instrument complexity. Flow-injection-MS/MS requires a single pump to deliver the solvent flow stream into the source of the mass spectrometer. In the case of LC-MS/MS a second pump is required to generate the binary solvent gradient, and an LC column is inserted into the flow stream line. Highly robust LC-MS/MS assays have been reported from the NBS laboratories in Austria [34], Italy [8], Illinois [9], Taiwan [10], Washington (C. R. Scott, WORLDSymposium, San Diego 2018) and Connecticut (A. Manning, APHL Newborn Screening Symposium, New Orleans 2017).

## 7. Concluding Remarks

In summary, MS/MS and DMF-F platforms are both highly suitable for NBS of Pompe disease and MPS-I. The workflows, including the number of manual steps and the incubation times, have been summarized, in detail, so that each NBS laboratory can decide which method is most adaptable to their program. The DMF-F platform offers a simplified assay instrument compared to MS/MS and is expected to require less maintenance. Precision studies carried out by the CDC in an identical setting and with identical samples show that MS/MS provides improved assay precision over DMF-F. MS/MS offers additional flexibility for the expansion of NBS programs to include additional LSDs and diseases where a fluorimetric assay is not possible. The majority of the attenuated enzymatic activity screen positives obtained by DMF-F and MS/MS are false positives, and second tier tests will be required either by the NBS laboratory or the follow-up laboratory. Post-test statistical analysis (such as CLIR) is expected to be useful as well. A number of LSDs have both pseudodeficiencies and late-onset variation, and this presents a special challenge to families and medical follow-up centers. Nevertheless, NBS for a subset of LSDs can lead to better health outcomes for affected patients.

## Figures and Tables

**Table 1:** Summary of the workflows for mass spectrometry (MS/MS) and digital microfluidics fluorimetry (DMF-F) lysosomal storage diseases (LSD) newborn screening (NBS) assays with dried blood spots (DBS). Hands-on time per step is given for the MS/MS assay only.

	Assay Step	Number of Liquid Transfers Per 96 Samples ^1^
**DMF-F**	Transfer enzyme extraction solvent to 96 well plate of DBS punches, seal plate, shake at room temperature for 30 min	1 transfer with a 96-channel pipettor
	Load two microfluidics plates with filler fluid	2 transfers
	Load four calibrator solutions one by one to two microfluidics plates	8 transfers
	Load two wells to each of two microfluidics plates with stop buffer, one for each enzyme	4 transfers
	Load two wells to each of two microfluidics plates with enzyme assay cocktail, one for each enzyme	4 transfers
	Load samples to each of two microfluidics plates four at a time per column	2 transfers with a 48 channel pipettor
	Place microfluidics plates in plate reader	0
**MS/MS**	Remove single assay cocktail from storage and aliquot to 96-well plate containing DBS punches	1 transfer with a 96-channel pipettor, 10 min
	Seal plate and place in thermostated incubator/shaker for 3 h to overnight	0 transfers,
	Add quench solvent to each well	1 transfer with a 96-channel pipettor, 5 min
	Transfer an aliquot of each well to a deep-well, 96-well plate	1 transfer with a 96-channel pipettor, 5 min
	Add ethyl acetate to each well	1 transfer with a 96-channel pipettor, 1 min
	Add water to each well	1 transfer with a 96-channel pipettor, 1 min
	Centrifuge for 5 min at room temperature	0 transfers, 5 min
	Transfer an aliquot of upper ethyl acetate layer to a shallow-well, 96-well plate	1 transfer with a 96-channel pipettor, 5 min
	Remove solvent with a multi-jet air dryer in fume hood	0 transfers, 10 min
	Add MS/MS infusion solvent to each well, seal plate and place on shaker at room temperature for 10 min	1 transfer with a 96-channel pipettor, 6 min
	Place 96-well plate in autosampler of MS/MS instrument	0 transfers, 5 min

^1^ The DMF-F cartridge accepts 48 samples, and thus two are needed for 96 samples. The MS/MS assay uses 96-well plates. The number of liquid transfers is based on two enzyme assays per sample (i.e. Pompe and MPS-I). The MS/MS assay uses a standard 96-channel pipet (Rainin Liquidator or the equivalent). This could be used for some, but not all, of the steps for DMF-F. For NBS of Pompe disease and MPS-I (relatively fast lysosomal enzymes), same day results are obtained using DMF-F, whereas the MS/MS assay results are obtained as early as the morning of day 2.

**Table 2:** NBS laboratory screen positive rates for Pompe disease.

NBS Laboratory	NBS Method	Current Cutoff	Number of Below Cutoff Samples	Number of below Cutoff Samples Per 100,000 Newborns	Source of Data
New York	MS/MS	15% of mean GAA activity	147 per 660,000	22	C. Stevens, J. Orsini, New York DOH [15]
Washington	MS/MS	15% of mean GAA activity	9 per 45,000	20	[3,15]
Ohio	MS/MS	8.5% of mean GAA activity	7 per 149,000	5 (32 if used 15% cutoff)	R. Hage, Ohio DOH [15]
Illinois	MS/MS	18% of mean GAA activity	139 per 219,000	63 (14 if use 15% cutoff)	R. Shao, Illinois DOH; [9,15]
Pennsylvania	MS/MS	19% of mean GAA activity	18 per 104,000	18	Pennsylvannia DOH [15]
Wisconsin	MS/MS	15% of mean GAA activity	1 per 8,250	12	M. Baker, Wisconsin DOH [15]
Tennessee	MS/MS	15% of mean GAA activity	2 per 12,279	16	G. Dizikes, Tennessee DOH [15]
Kentucky	MS/MS	Footnote 1	2 per 55,000	4	[14]
New York City	MS/MS	15% of mean GAA activity	6 per 18,105	33	[16]
Italy (Veneto Region)	MS/MS	19% of mean GAA activity	5 per 44,000	11 per 100,000	[17]

^1^ Uses CLIR tools [14] rather than cutoffs (see main text).

**Table 3:** NBS laboratory screen positive rates for MPS-I.

NBS Laboratory	NBS Method	Current Cutoff	Number of Below Cutoff Samples	Number of below Cutoff Samples Per 100,000 Newborns	Source of Data
New York	MS/MS	10% of IDUA activity	7 per 32,000	22 (20 if used 7.2%) ^2^	(J. Orsini, New York DOH) [15]
Washington	MS/MS	10% of IDUA activity	6 per 45,000	13 (7 if used 7.2%)	[3,15]
Ohio	MS/MS	7.2% of IDUA activity	19 per 149,000	13	(R. Hage, Ohio DOH) [15]
Illinois	MS/MS	28% of IDUA activity	151 per 219,000	69 (11 if used 7.2%)	(R. Shao, Illinois DOH) [9,15]
Kentucky	MS/MS	Footnote 1	1 per 55,000	2	[14]
New York City	MS/MS	15% of mean IDUA activity	13 per 35,816	36 ^2^	[16]
Chinese Foundation of Health (Taiwan)	MS/MS	7.2% of IDUA activity	5 per 93,000	6	(H-C. Liao, Chinese Foundation of Health) [15]
National Taiwan University Hospital (Taiwan)	MS/MS	15% of IDUA activity	2 per 62,562	3 (3 if use 7.2%)	(P. Hwu, National Taiwan University Hospital) [15]
Italy (Veneto region)	MS/MS	19% IDUA activity	8 per 44,000	18 per 100,000	[17]

^1^ Uses CLIR tools [14] rather than cutoffs (see main text). ^2^ The New York NBS laboratory is currently using a cutoff of 8% of mean IDUA activity (personal communication with J. Orsini, Wadsworth Center).

**Table 4:** Published newborn screening assays for LSDs using DBS ^1^.

LSD	MS/MS Assay	DMF-F Assay
Ceroid lipofuscinoisis I	[24] ^2^	
Ceroid lipofuscinoisis II	[4] ^1^	
Fabry	[3]	[2]
Gaucher	[3]	[2]
Krabbe	[3]	
Metachromatic Leukodystrophy	[30] ^2,3^	
MPS-I	[3]	[2]
MPS-II	[4]	[33]
MPS-IIIA	[13] ^2^	
MPS-IIIB	[4]	
MPS-IVA	[4] ^2^	
MPS-VI	[4] ^2^	
MPS-VII	[4]	
Niemann-Pick-A/B	[3]	
Niemann-Pick-C	[31] ^2,4^	
Pompe	[3]	[2]
Wolman Disease	[23]	

^1^ All assays measure lysosomal enzymatic activity unless stated otherwise. ^2^ Requires LC-MS/MS. ^3^ Sulfatide accumulation in DBS. ^4^ Bile acid derivative accumulation in DBS.

## Supplementary Materials

The following are available online at https://www.mdpi.com/2409-515X/5/1/1/s1.
