# Supplementary material for: Newborn Screening for Lysosomal Storage Disorders: Methodologies for Measurement of Enzymatic Activities in Dried Blood Spots
Source: Int J Neonatal Screen. 2018 Dec 21;5(1):1. doi: 10.3390/ijns5010001 (PMC6448570; doi:10.3390/ijns5010001)
Supplement: Supplementary file 1 [file IJNS-05-00001-s001.pdf]

Supplemental Material

Newborn Screening Quality Assurance Program

Lysosomal Storage Disorder Quality Control (LSDQC) Specimen Certification

Set 2 – July 2018

Analysis Method: Digital Microfluidic Fluorescence

Expiration Date: March 2020
Material Storage: -20°C ± 10°C
Units of Measure: µmol/hr/L blood

QC Lot Number: A1708

Analyte	Mean Activity Level	95% LL	95% UL
Acid α-Glucosidase (GAA)	0.96	0	5.48
α-L-Iduronidase (IDUA)	1.32	0	4.71
α-Galactosidase (GLA)	2.84	0	9.93
β-Glucocerebrosidase (ABG)	1.59	0	5.00

Expiration Date: March 2020
Material Storage: -20°C ± 10°C
Units of Measure: µmol/hr/L blood

QC Lot Number: B1708

Analyte	Mean Activity Level	95% LL	95% UL
Acid α-Glucosidase (GAA)	2.00	0.34	3.66
α-L-Iduronidase (IDUA)	2.85	0	7.65
α-Galactosidase (GLA)	4.41	0	9.97
β-Glucocerebrosidase (ABG)	2.04	0	5.40

Expiration Date: March 2020
Material Storage: -20°C ± 10°C
Units of Measure: µmol/hr/L blood

QC Lot Number: C1708

Analyte	Mean Activity Level	95% LL	95% UL
Acid α-Glucosidase (GAA)	14.64	7.23	22.06
α-L-Iduronidase (IDUA)	14.01	8.21	19.81
α-Galactosidase (GLA)	26.62	16.12	37.12
β-Glucocerebrosidase (ABG)	6.54	3.62	9.45

Note: The values provided in the above tables are for reference use only. The mean value and confidence limits (CL) are determined by CDC for each Quality Control (QC) lot. Each participating laboratory must establish its own mean values and CL for its test method with these QC materials. Temporary estimates of mean values and CL can be determined after 10 successive, independent measurements. *Slazyk WE, Hannon WH. Quality Assurance in the newborn screening laboratory. In: Therrell BL Jr, editor. Laboratory methods for neonatal screening. Washington (DC): American Public Health Association, 1993:23-46.*

Figure S1. CDC Certificate Report 1. Certificate report (DMF-F) posted by the CDC for multiple analysis of their QC standards. A is base, B is low, C is medium, and D is high. Obtained from the CDC public NBS web portal. Note that the report has a truncated value for the 95% LL at 0 mmol/h/L since the 95% lower confident limit gives a negative enzymatic actiity value (which is acceptable since the assay response for the complete assay with DBS may actual be less than that from the blank).

Newborn Screening Quality Assurance Program

Lysosomal Storage Disorder Quality Control (LSDQC) Specimen Certification

Set 2 – July 2018

Analysis Method: Digital Microfluidic Fluorescence

Expiration Date: March 2020
Material Storage: -20°C ± 10°C
Units: µmol/hr/L blood

QC Lot Number: D1708

Analyte	Mean Activity Level	95% LL	95% UL
Acid α-Glucosidase (GAA)	24.65	17.74	31.55
α-L-Iduronidase (IDUA)	25.98	17.64	34.33
α-Galactosidase (GLA)	50.34	32.86	67.81
β-Glucocerebrosidase (ABG)	10.15	5.31	14.99

Note: The values provided in the above tables are for reference use only. The mean value and confidence limits (CL) are determined by CDC for each Quality Control (QC) lot. Each participating laboratory must establish its own mean values and CL for its test method with these QC materials. Temporary estimates of mean values and CL can be determined after 10 successive, independent measurements. *Slazyk WE, Hannon WH. Quality Assurance in the newborn screening laboratory. In: Therrell BL Jr, editor. Laboratory methods for neonatal screening. Washington (DC): American Public Health Association, 1993:23-46.*

Lysosomal Storage Disorder Quality Control (LSDQC) Specimen Certification

Set 2 – July 2018

Analysis Method: Flow Injection Analysis MS/MS Non-kit

Expiration Date: March 2020
Material Storage: -20°C ± 10°C
Units of Measure: µmol/hr/L blood

QC Lot Number: A1708

Analyte	Mean Activity Level	95% LL	95% UL
Galactocerebrosidase (GALC)	0.33	0.30	0.37
Acid α-Glucosidase (GAA)	0.15	0.11	0.19
α-L-Iduronidase (IDUA)	0.12	0.11	0.14
α-Galactosidase (GLA)	1.11	0.87	1.35
β-Glucocerebrosidase (ABG)	0.68	0.54	0.83
Acid Sphingomyelinase (ASM)	0.08	0.06	0.11

Expiration Date: March 2020
Material Storage: -20°C ± 10°C
Units of Measure: µmol/hr/L blood

QC Lot Number: B1708

Analyte	Mean Activity Level	95% LL	95% UL
Galactocerebrosidase (GALC)	0.67	0.63	0.72
Acid α-Glucosidase (GAA)	0.87	0.76	0.98
α-L-Iduronidase (IDUA)	0.60	0.55	0.66
α-Galactosidase (GLA)	1.78	1.52	2.05
β-Glucocerebrosidase (ABG)	1.26	1.01	1.51
Acid Sphingomyelinase (ASM)	0.22	0.18	0.26

Note: The values provided in the above tables are for reference use only. The mean value and confidence limits (CL) are determined by CDC for each Quality Control (QC) lot. Each participating laboratory must establish its own mean values and CL for its test method with these QC materials. Temporary estimates of mean values and CL can be determined after 10 successive, independent measurements. *Slazyk WE, Hannon WH. Quality Assurance in the newborn screening laboratory. In: Therrell BL Jr, editor. Laboratory methods for neonatal screening. Washington (DC): American Public Health Association, 1993:23-46.*

Newborn Screening Quality Assurance Program

Lysosomal Storage Disorder Quality Control (LSDQC) Specimen Certification

Set 2 – July 2018

Analysis Method: Flow Injection Analysis MS/MS Non-kit

Expiration Date: March 2020
Material Storage: -20°C ± 10°C
Units of Measure: µmol/hr/L blood

QC Lot Number: C1708

Analyte	Mean Activity Level	95% LL	95% UL
Galactocerebrosidase (GALC)	3.52	2.74	4.29
Acid α-Glucosidase (GAA)	5.66	3.66	7.67
α-L-Iduronidase (IDUA)	4.38	3.23	5.53
α-Galactosidase (GLA)	7.44	6.59	8.28
β-Glucocerebrosidase (ABG)	5.88	4.37	7.40
Acid Sphingomyelinase (ASM)	1.21	1.06	1.37

Expiration Date: March 2020
Material Storage: -20°C ± 10°C
Units of Measure: µmol/hr/L blood

QC Lot Number: D1708

Analyte	Mean Activity Level	95% LL	95% UL
Galactocerebrosidase (GALC)	6.68	5.88	7.48
Acid α-Glucosidase (GAA)	9.02	7.80	10.25
α-L-Iduronidase (IDUA)	8.04	5.87	10.21
α-Galactosidase (GLA)	13.52	12.19	14.84
β-Glucocerebrosidase (ABG)	10.91	8.99	12.84
Acid Sphingomyelinase (ASM)	2.04	1.69	2.39

Note: The values provided in the above tables are for reference use only. The mean value and confidence limits (CL) are determined by CDC for each Quality Control (QC) lot. Each participating laboratory must establish its own mean values and CL for its test method with these QC materials. Temporary estimates of mean values and CL can be determined after 10 successive, independent measurements. *Slazyk WE, Hannon WH. Quality Assurance in the newborn screening laboratory. In: Therrell BL Jr, editor. Laboratory methods for neonatal screening. Washington (DC): American Public Health Association, 1993:23-46.*

Figure S2. CDC Certificate Report 2. Same as Figure S1 but for MS/MS.
